# Interfacial engineered superelastic metal-organic framework aerogels with van-der-Waals barrier channels for nerve agents decomposition

**DOI:** 10.1038/s41467-023-37693-5

**Published:** 2023-04-13

**Authors:** Zishuo Yan, Xiaoyan Liu, Bin Ding, Jianyong Yu, Yang Si

**Affiliations:** 1 State Key Laboratory for Modification of Chemical Fibers and Polymer Materials, College of Textiles, Donghua University, Shanghai, 201620 China; 2 Innovation Center for Textile Science and Technology, Donghua University, Shanghai, 200051 China

**Keywords:** Porous materials, Synthesis and processing

## Abstract

Chemical warfare agents (CWAs) significantly threaten human peace and global security. Most personal protective equipment (PPE) deployed to prevent exposure to CWAs is generally devoid of self-detoxifying activity. Here we report the spatial rearrangement of metal-organic frameworks (MOFs) into superelastic lamellar-structured aerogels based on a ceramic network-assisted interfacial engineering protocol. The optimized aerogels exhibit efficient adsorption and decomposition performance against CWAs either in liquid or aerosol forms (half-life of 5.29 min, dynamic breakthrough extent of 400 L g^−1^) due to the preserved MOF structure, van-der-Waals barrier channels, minimized diffusion resistance (~41% reduction), and stability over a thousand compressions. The successful construction of the attractive materials offers fascinating perspectives on the development of field-deployable, real-time detoxifying, and structurally adaptable PPE that could be served as outdoor emergency life-saving devices against CWAs threats. This work also provides a guiding toolbox for incorporating other critical adsorbents into the accessible 3D matrix with enhanced gas transport properties.

## Abstract (web summary)

Personal protective equipment used to prevent exposure to chemical warfare agents are devoid of detoxifying activity. Here, the authors report MOF aerogels via a hydrogen bonding-assisted interfacial engineering strategy for nerve agent decomposition.

## Introduction

Chemical warfare agents (CWAs) continue to pose significant threats to human life and national security^1^. The potential utilization of CWAs in combat operations or terrorist attacks remains in the complicated international environment, despite their global prohibition and destruction by the Organization for the Prohibition of Chemical Weapons (OPCW)^2^. Chemically and toxicologically, organophosphorus nerve agents such as sarin, soman, and VX are extremely lethal that can phosphorylate the enzyme acetylcholinesterase, resulting in depression of the central nervous system^3^. Current defense technology for protecting emergency personnel against CWAs mainly depends on impregnated active-carbon-containing protective clothing^4^. Nevertheless, it suffers from secondary emission after saturation and requires final disposal^5^, presenting a pressing need to develop advanced personal protective equipment (PPE) capable of reliable adsorption and self-detoxification of CWAs.

An optimal self-detoxifying PPE requires two crucial features: tailored porous traps and interconnected hierarchical channels, serving as specific space for capturing CWAs with less diffusion resistance, and substantial open active sites, which could decompose them into nontoxic products simultaneously. Among the recently explored materials, zirconium hydroxide, metal oxides, metal-organic frameworks (MOFs), and polyoxometalates, have shown tremendous promise for the real-time decontamination of nerve agents^6–9^. The toxic chemicals could be adsorbed onto the materials and decomposed by hydrolysis of labile phosphate ester bonds^10^. Notably, in comparison with other candidates, the ultrahigh surface areas, favorable porosity, chemical versatility, and coordinatively unsaturated metal sites further endow the zirconium-based MOFs with infinite opportunities for ideal self-detoxifying PPE against nerve agents^11^. However, the inherent limitation of their natural powder form significantly hampers the development and application in emergency medical services, which is not the preferred configuration for PPE^12^. The assembly of MOF-derived textiles by coating^13^, layer-by-layer^14^, and hot pressing^15^ has proven to be a reliable approach to utilize the desired characteristics of MOFs in protective clothing. Despite the enhanced practical performance of these composites, their tightly stacked two-dimensional structure still leads to short functional paths and limited air permeability, which will result in reduced degradation capacity and rate^16^. In addition, the composites possess considerable interfacial area between components. Interfacial engineering is of paramount importance for achieving reliable adhesion to avoid extreme situations such as insufficient interactions or rigidified objects that could affect structural stability and durability^17^. Therefore, the challenge is the three-dimensional reconstruction of the composites and precisely engineering the MOFs/nanofibers interface in the process to obtain a continuous monolith capable of sufficient mechanical robustness and high air permeability, without compromising the decontamination performances of MOFs against nerve agents.

Here, we propose a facile protocol to tailor interfacial coupling of MOFs/nanofibers composites by introducing trimethoxymethylsilane (MTMS)-derived silica sol. Employing molecular dynamics (MD) and density functional theory (DFT) simulations, we show that hydroxyl terminations on the silica sol offer hydrogen bonding with the MOFs and ceramic nanofibers, leading to strong adhesion and enhanced mechanical stability of the macroscopic composites. Specifically, we combine this hydrogen bond anchoring strategy with the freeze-drying technique to shape functional MOFs and structural SiO_2_ nanofibers into superelastic hierarchical aerogels (MNAs). The MOFs are distributed in the interconnected channels of 3D aerogels and preserve their porosity, crystallinity, and accessible chemically active sites, providing a suitable platform for physical trapping and chemical catalyst for nerve agent treatment. Moreover, the intruding ceramic constituents in the interconnected channels afford van der Waals barriers that facilitate preferential nerve agent adsorption in open MOF sites. The as-prepared MNAs exhibit integrated properties of high porosity, low filtration resistance, excellent adsorption and decontamination efficacy, and complete recovery from large deformations. MNAs are the most attractive options for self-detoxifying PPE that could be deployed as outdoor emergency life-saving devices against CWAs threats either in liquid or aerosol forms.

## Results

### The interfacial engineering strategy used to design MNAs

**Fig. 1:**
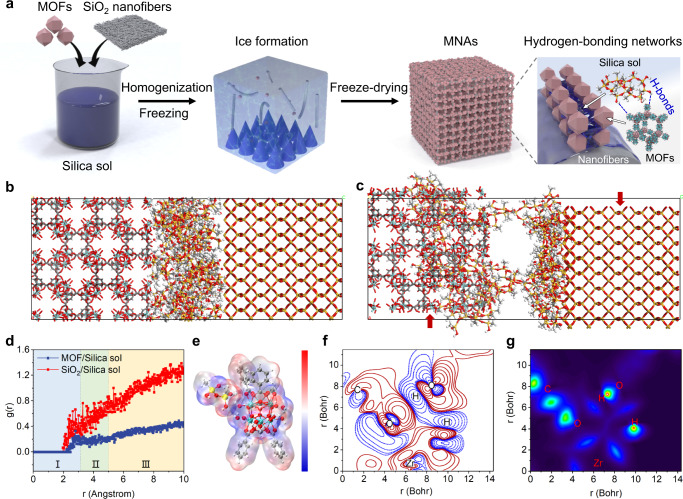
Design strategies for interfacial engineered MOF aerogels. **a** Fabrication procedures of the interfacial engineered MNAs. Simulations of in-situ alignment of silica sol chain before (**b**) and after (**c**) confined shear. The corresponding radial distribution functions (**d**). **e** ESP of the optimized MOF/silica sol complex is drawn over the range of −0.03 to 0.03. The electron density difference isosurfaces (**f**) and IGMH-based *δ*g function map (**g**) on the defined plane in the complex. The red and blue lines of isosurfaces stand for the regions of increased and decreased electron density, respectively. The map is colored according to a BWR (blue-green-red) scheme over the range of 0–0.5. Source data are provided as a Source Data file.

We designed the MNAs according to three crucial considerations: (i) the nanofibers must be assembled into an elastic and open-cell skeleton; (ii) MOFs must be well-anchored in the porous monolith; (iii) the contaminated fluid must be detoxified into nontoxic products effectively and rapidly. The first two requirements were enabled by phase separation-induced structural shaping and hydrogen bonding-assisted interfacial engineering. The stable Si-O-Si networks in the binder and hydrogen-bonding interactions at the interface with other components were formed synchronously by incorporating silica sol in the aerogels. Figure 1a depicts the synthesis procedures for the excellent self-detoxifying aerogel. Specifically, the SiO_2_ nanofibers that serve as the rigid support in the aerogel were fabricated by sol-gel electrospinning^18^. The as-prepared electrospun nanofibrous membranes and MOFs (Supplementary Fig. 1) were then homogenized in the silica sol to prepare a well-dispersed suspension. Subsequently, the dispersion was frozen in liquid nitrogen and lyophilized into MNAs. The principle of the mechanically favorable composite aerogels was based on the in-situ condensation of the hydrolyzed silica sol wrapped between the nanofibers and MOFs, which led to the formation of elastic Si-O-Si cross-linking networks along with hydrogen-bonding networks, contributing to cementation of the adjacent solid components (Supplementary Fig. 2).

To determine whether hydrogen-bonding networks could promote the strong interfacial connections of the components in MNAs, we first conducted MD and DFT simulations to demonstrate the intrinsic stability of Zr-based MOFs, silica sol, and silica nanofibers ternary system. The dynamic response of the silica sol, confined in Couette flow geometries, under shear conditions between crystalline MOFs and amorphous silica walls (Supplementary Fig. 3) was exhibited in Fig. 1b, c^19^. The Lennard-Jones potential was adopted for the interfacial connections between the simulated walls and organic molecules^20^. The left and right walls were subjected to constant forces at a rate of 0.05 Å ps^−1^ in the opposite direction along the Z axis. Periodic boundary conditions were applied in the other two directions to reduce the edge effects^21^. We verified that the silica sol attained a stable state after the 50 ps simulation by tracking the changes in system energies and wall stress. As shown in the final snapshot (Fig. 1c), the silica sol was contained between the walls and formed into a typical configuration known as a wall-induced layering structure^22^. The silica sol adhered to the adjacent walls and some of the chains slipped into the interior of the confined layers, which was driven by the affinity with the walls. To provide insight into the interaction mechanism, we carried out the radial distribution function (RDF) analysis for the nanocomposite systems, which refers to the probability to find silica sol at a certain distance *r* specified by the reference walls^23^. The derived RDF for the MOF/silica sol and SiO_2_/silica sol deployed during the confined shear simulations are presented in Fig. 1d. The peak value for MOF/silica sol is 0.33 at 2.79 Å, corresponding to the formation of hydrogen bond (Region I: *r* < 3.1 Å). There are no other significant peaks outside Region I, where the interactions are mainly governed by the van der Waals bond (Region II: 3.1 Å < *r* < 5 Å) and electrostatic interaction (Region III: *r* > 5 Å)^24^. Additionally, a similar result is obtained for the SiO_2_/silica sol in Fig. 1d, exhibiting higher g(r) values that indicate a greater potential for hydrogen bond formation compared to the MOF/silica sol, which could prevent excessive penetration into the MOF pore structure^25^. Such simulations of the MOF/silica sol/SiO_2_ ternary system support the preliminary finding that the hydrogen bonds engineered in the interface improve the interfacial interactions.

Taking MOF-808 and silica sol as the model system, the intermolecular interaction is thoroughly investigated by DFT calculations. Details of the models and simulations are described in Supplementary Note 4. The results showed that the silica sol binds onto the surface of MOFs through hydrogen bonds with the Gibbs free energy change of −23 kJ mol^−1^, achieving interphase cross-linking networks in the MNAs (Supplementary Figs. 4, 5)^26^. As shown in Fig. 1e, the electrostatic potential (ESP) of the molecules was employed to demonstrate the formation of the hydrogen bond in the system^27^. The red area of the H atom indicates the positive value of ESP, while the blue area of the O atom denotes the negative value of ESP. The attraction of positively charged sites to negative sites tends to favor the formation of hydrogen bonds. Furthermore, we precisely checked the non-covalent interactions in the plane defined by O1, H2 atoms of MOF, and H1 atom of silica sol (Supplementary Fig. 5)^28^. Electron density difference map^29^ and independent gradient model based on Hirshfeld partition (IGMH)^30^ were introduced to explain the non-covalent interactions between MOF and silica sol in the two-dimensional plane. Figure 1f and Supplementary Fig. 6 presented the transfer of electrons resulting from the bond formation, where the red and blue lines stand for the regions of increased and decreased electron density, respectively. The electron densities near the H and O atoms that formed hydrogen bonds were markedly reduced as a result of the Pauli repulsion effect. Simultaneously, the regions of increased electron densities between the H and O atoms were observed, suggesting that the non-covalent interactions were also based on the electron delocalization effect between H and O atoms^31^. To effectively exhibit the non-covalent interactions, we mapped the two-dimensional IGMH function of the defined plane (Fig. 1g)^32^. The blue isosurfaces between MOF and silica sol were identified as the hydrogen bond interaction regions, which coincides well with the analysis of the electron density difference maps^33^. According to the above simulation results, hydrogen bonds play a crucial role in stabilizing the interface of MOF-808 and silica sol, thus promoting the robust formation of the MNAs.

### Fabrication and characterization of MNAs

**Fig. 2:**
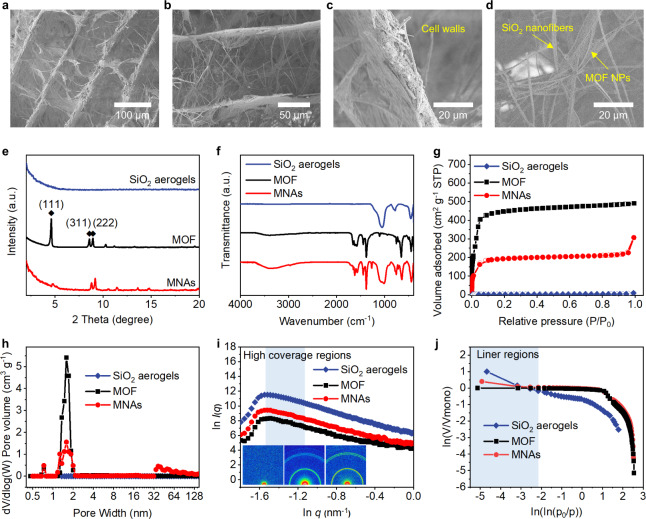
Structural characterizations of MNAs. **a**–**d** Morphologies of MNAs with lamellar MOFs/nanofibers skeleton at various magnifications. **e** XRD, **f** FT-IR spectra, **g** Nitrogen adsorption-desorption isotherms, and **h** pore size distribution based on the DFT models of relative materials. Fractal analysis calculated from SAXS (**i**) and nitrogen adsorption isotherms (**j**), the insets show the SAXS 2D patterns. Source data are provided as a Source Data file.

Typical scanning electron microscope (SEM) and transmission electron microscopy (TEM) images of the MNAs are shown in Fig. 2a–d and Supplementary Fig. 7, which have an aligned lamellar morphology bearing MOFs/nanofibers skeleton and interconnected pores. The MOF NPs were found to be firmly positioned on the surface of fibers when the cellular walls were magnified, suggesting the robust formation of the reliable interface. Phase separation brought on by solvent crystallization during the freeze-drying process could be the governing factor in the construction of the cellular structure. In the process of freezing a homogenous dispersion, the solids were repelled from the growing ice crystals and then collected between the adjacent icicles, leading to a well-defined composite skeleton. Simultaneously, the silica sol accumulated on the MOFs/nanofibers’ surface, introducing strong adhesion dominated by the hydrogen bonds between them. Additionally, energy-dispersive X-ray spectroscopy was used to demonstrate the uniform distribution of MOF particles in MNAs (Supplementary Fig. 8).

The microstructures of the composite aerogels and interactions among the components were elucidated by X-ray diffraction (XRD), Fourier transform infrared spectroscopy (FT-IR), and ^29^Si solid-state cross-polarization/magic-angle spinning nuclear magnetic resonance (CP/MAS NMR)^34^. As shown in Fig. 2e, the amorphous nature of the pure SiO_2_ nanofibrous aerogels was confirmed. The XRD pattern of pristine MOF-808 revealed the typical diffraction peak at 4.6° for the reflection plane of (111), along with smaller peaks at 8.6° and 9.0° that were ascribed to the crystal planes of (311) and (222)^35^. The XRD of MNAs appeared to be consistent with the original MOF-808, except for the low-angle reflection (111). The peak intensity (111) decreased in comparison to the higher angle reflections, indicating that silica sol entered the pore structure of the MOFs in MNAs during the fabrication^36^. It should be highlighted that the silica sol provides strong interphase compatibility with MOF-808, which is in good agreement with the outcomes anticipated by the interfacial hypothesis. This conclusion was strengthened by the differences in the FT-IR spectra of the relative samples (Fig. 2f and Supplementary Table 1). The asymmetric stretching vibration peak of Si-O-Si at 1015 cm^−1^, symmetric stretching vibrations of Si-O at 758 and 443 cm^−1^, and the stretching vibration peak of -OH at 3381 cm^−1^ were observed in MNAs, with an obvious shift to the lower wavenumber compared to those of SiO_2_ aerogels and pure MOF-808^37^. This revealed a robust formation of hydrogen bonds in the composite aerogels. In addition, as shown in Supplementary Fig. 9, compared with the SiO_2_ nanofibrous aerogels, the ^29^Si CP/MAS NMR spectra of the MNAs revealed a chemical shift of Si-O with the introduction of MOF-808, suggesting the formation of the hydrogen bonds in MNAs^38^. Overall, XRD, FT-IR, and CP/MAS NMR demonstrated the adhesion of the MOFs to the SiO_2_ aerogels by introducing a hydrogen bond-regulated silica sol interface between them^39^.

After identifying the morphologies and chemical structures of the obtained materials, we subsequently studied their porous characteristics, as shown in Fig. 2g and Supplementary Table 2. The porosity and surface area of the above materials were elucidated using N_2_ adsorption-desorption isotherm measurements at 77 K. The isotherm of MNAs implied a type I/II character with sharp adsorption at a low relative pressure (*P*/*P*_0_ < 0.01) and a steep uptake at a high relative pressure (*P*/*P*_0_ > 0.95), which was attributed to the abundant micropores filling of MOFs and stacked macropores sorption on the cell walls^40^. The Brunauer−Emmett−Teller surface area of MNAs was estimated to be 600 m^2^ g^−1^. The result falls between those of the MOF-808 (1661 m^2^ g^−1^) and SiO_2_ nanofibrous aerogels (5 m^2^ g^−1^). The pore size distribution evaluated with density functional theory (DFT) indicated the formation of the hierarchical structure of micro-meso-macropores in the MNAs (Fig. 2h). To further illustrate the multimodal porosity and structural fluctuations of the MNAs, we performed the small-angle X-ray scattering (SAXS) technique. The insets of Fig. 2i exhibited two-dimensional SAXS patterns of SiO_2_ aerogels, MOFs, and MNAs, respectively. There is an ordered structure of MOFs in MNAs because of the apparent scattering ring. The areas of scattering patterns under the same intensity could indicate the number of interior porosities, which coincides well with the results assessed by the nitrogen adsorption method^41^. We employed the parameter of fractal analysis (*D*), which is computed by integrating SAXS (Fig. 2i) and the nitrogen sorption method (Fig. 2g), to provide precise details on the geometric structural properties of porous structures in the samples^42^. The fractal dimension of SAXS (D_s_) is quantified by the slope of the logarithmic curve, while the fractal size of the latter (*D*_N_) is determined using the apparent linear slope of the high-coverage region according to the Frenkel–Halsey–Hill equation^43^. The value of *D*_N_ is notably smaller than *D*_S_ due to the inaccessibility of the nitrogen to the closed pores, while *D*_s_ is associated with both open and closed pores since it is based on the difference in electron density in the two-phase system. The *D* values of MNAs were 2.93 (*D*_S_) and 2.87 (*D*_N_), respectively, indicating a typical surface fractal feature and the dominance of open pores in the composite aerogels. The (*D*_S_-*D*_N_)/*D*_S_ (percentage of closed pores) value of MNAs was slightly increased compared to MOFs by about 2 percent. Pores in MOFs are partially blocked when silica sol is introduced, yet it is acceptable. The above investigations, along with the theoretical predictions, corroborate the utility of interfacial engineering based on the well-defined hydrogen-bonding networks in the resultant composite aerogels.

### Stability and gas-permeation behavior

**Fig. 3:**
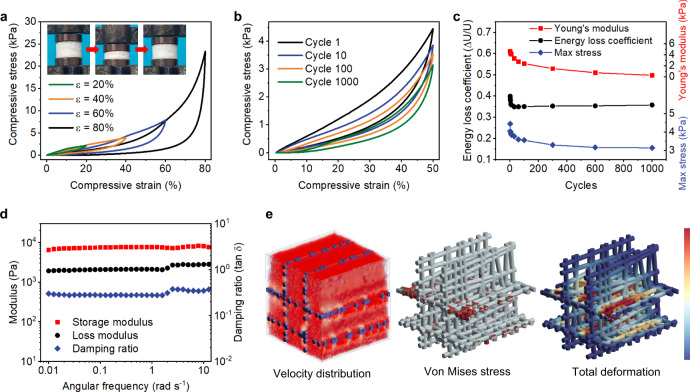
Mechanical properties and permeation. **a** Compressive stress-strain curves of MNAs. The insets show the photographs of the aerogel under a cycle of compression and release. A 1000-cycle compression fatigue test (**b**) and the resultant young’s modulus, energy loss coefficient, and maximum stress (**c**). **d** The frequency dependence of damping ratio, storage modulus, and loss modulus. **e** Simulated distributions of airflow, von Mises stress, and total deformation in the fluid-solid coupling process. The distributions are normalized and colored over the range of 0–1. Source data are provided as a Source Data file.

Compared to conventional particle-based materials, which are structurally unstable, the tailored interfacial engineered MNAs exhibited robust mechanical characteristics and could completely spring back to their original position without cracking once the stress is removed (insets in Fig. 3a)^44^. We performed a series of compression experiments that the samples were applied continuous axial stress until the desired strain was attained. The compression curves (σ–ε) displayed classical closed loops with three specific regions, which are typical of the cellular structure: an initial linear elastic region (ε < 10%), a subsequent plateau region (20% < ε < 60%), and a final densification region (ε > 60%) with an abrupt increase in stress^45^. The recovery force always remains positive, revealing the elastic deformation of MNAs even under large strain. Moreover, the mechanical durability of stress transfer in aerogels is crucial in long-term deployment. As shown in Fig. 3b, c, the material was slightly damaged with 5% irreversible deformation and preserved 71% of the beginning maximum stress after 1000 loading-unloading cycles of compression, indicating that the interconnected porous skeleton buckled and recovered without breaking. In addition, dynamic mechanical analysis (DMA) was employed to elucidate the dynamic compressive investigations of the aerogels. With the test angular frequency ranging from 0.01 to 10 Hz, the properties such as storage and loss modulus and damping ratio of the samples generally stayed constant, revealing the remarkable viscoelasticity of MNAs. The above-observed stabilities highlight that the MOF particles in the composite aerogels are efficiently anchored by introducing strong hydrogen-bonding networks.

Moreover, the stacked particle-based materials inevitably lead to remarkable mass transfer resistance, limiting the deeper penetration to the adsorbent^46^. In contrast, the spatial interconnected porous architecture of MNAs enables enhanced transportation of reactants in the pore channels, and the highly dispersed 0D MOFs in the 3D skeleton facilitate the adequate exposure of nerve agents to the coordinatively unsaturated Zr sites during the diffusion process. To highlight the advanced structure of 3D aerogels, we fabricated the MOF-based 2D nanofibrous membranes and characterized the pressure drops of the relevant samples. The MNAs possessed a lower pressure drop than that of nanofibrous membranes at the same airflow velocity (220 and 375 Pa, respectively). The 3D finite element model based on MNAs was constructed and the seepage-stress field coupling was established in the obtained continuous porous medium^47^. As shown in Fig. 3e, after loading an airflow at a constant rate of 1 m/s upon the Z axis of the structural model, we monitored the velocity distribution of fluid, as well as the von Mises stress and total deformation of the solid resulting from the fluid pressure^48^. The dominant red color in the airflow field suggested that the velocity almost remained unchanged in the aerogels. Compared to the previous related studies on 2D materials, this phenomenon revealed that the lamellar nanofibrous skeleton could facilitate the diffusion of fluid with less resistance^49^. There was a strong coupling between fluid and solid that caused a complicated mechanical response. The values of von Mises stress and total deformation were larger in the cross-section perpendicular to the direction of airflow, and tended to decrease as depth was increased. Overall, benefiting from the elastic cell walls, the mechanical response could be handled in the dynamic decontamination process.

### DMMP adsorption and degradation

**Fig. 4:**
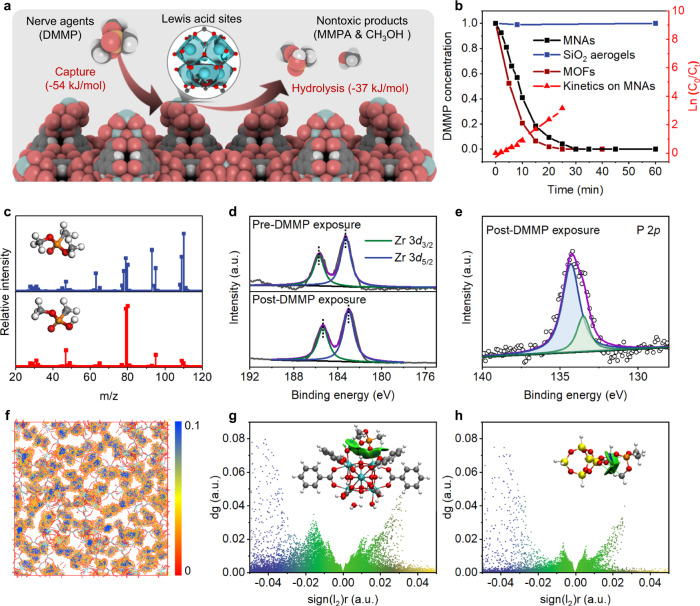
Adsorption and hydrolytic degradation reaction. **a** Schematic illustration of the capture and hydrolysis effects of the MOF surface in MNAs. **b** Concentration curves of DMMP and detoxification kinetics. **c** MS spectra of DMMP and MMPA. XPS spectra of Zr 3*d* (**d**) and P 2*p* (**e**) on MNAs. **f** MD simulation of DMMP density distribution in MOFs. Non-covalent interactions of DMMP/MOFs (**g**) and DMMP/SiO_2_ (**h**) based on colored IGMH isosurfaces of *δ*g_inter_ = 0.001 a.u. Insets: relevant optimized structure of the complexes. Blue denoted strong attractions, including hydrogen bond, green indicated van der Waals interaction, and yellow represented strong repulsion, including steric hindrance. Source data are provided as a Source Data file.

Given the integrated properties of tailored tridimensional design, abundant MOF constitution, and robust mechanical stability, we explored the availability of the MNAs as spatial adsorption and catalytic materials against chemical warfare agents. Figure 4a provides a schematic description of the detoxification properties of the well-anchored MOFs on aerogels that enable the adsorption and hydrolysis of dimethyl methylphosphonate (DMMP), a sarin surrogate. From the mechanistic viewpoint, with the initial binding of the O atom of *sp*^2^ hybridization in DMMP to the coordinatively unsaturated Zr sites on the porous surface (Supplementary Fig. 10), the pentacoordinated phosphorus intermediate is obtained by nucleophilic addition of the Zr-OH group to the coordinated DMMP^36^. The intermediate is then decomposed by methanol elimination, leaving the organophosphorus product of methyl methylphosphonic acid (MMPA) bound to the MOFs (Supplementary Fig. 11)^50^. To clarify the potential of the materials as PPE, the MNAs were evaluated for catalytic activity in *N*-ethylmorpholine buffered solutions. Gas chromatography–mass spectrometry (GC–MS) was introduced to detect the extract (Fig. 4b, c). MNAs possessed stable and rapid adsorption and detoxification properties to DMMP, converting it into nontoxic products completely in 30 min. The kinetics of the reaction was assessed by plotting Ln(C_0_/C_t_) versus the detoxification time, where C_0_ represented the initial concentration, and C_t_ represented the residual concentration of DMMP. The rate constant (K) of detoxification was determined to be 0.131 min^−1^, and the half-life was calculated as 5.29 min using the formula 0.693 K^−1^^51^. To further improve the practicality of the MNAs used in PPE, the non-volatile alkali polymer (polyethyleneimine (PEI)) instead of the traditional aqueous solutions was introduced to the composite aerogels (Supplementary Fig. 12). Remarkably, the adsorption and catalytic performances of the obtained PEI-MNAs are comparable to the MNAs using the volatile *N*-ethylmorpholine solution, highlighting their potential to integrate MOFs and bases together into a porous monolith. The dynamic adsorption and decontamination of the MNAs under a 1ppm DMMP contaminated flow was also confirmed in Supplementary Fig. 13. The breakthrough extent for CWAs treatment with aerogels is 400 L g^−1^.

The variation of chemical states and electronic characteristics of the MNAs was examined after exposure to DMMP by using X-ray photoelectron spectroscopy (XPS) studies. Specifically, the Zr 3*d* peaks were slightly moved toward lower binding energies in Fig. 4d. After the exposure to DMMP, the Zr atom was less positively charged, suggesting the charge redistribution during the bond formation with the intermediate^52^. As shown in Fig. 4e, the high-resolution P 2*p* spectrum could be separated into two species corresponding to two types of phosphorus, which demonstrated the presence of pentacoordinated phosphorus intermediates bound to the MOF surface as well. These results are consistent with the DMMP hydrolysis process described above, which requires initial coordination with Lewis acid (coordinatively unsaturated Zr) rather than Bronsted acid (O-H) sites on the MOF surface^35^.

To clarify the adsorption behavior in detail, we investigated the structural configuration and optimized energies of the complexes formed by DMMP adsorption on MOF and SiO_2_ based on DFT simulations. According to the density distribution of DMMP in MOF-808, the secondary building units are identified as the preferential adsorption sites instead of 1,3,5-benzenetricarboxylate linkers (Fig. 4f)^53^. By using the IGMH analysis, the intermolecular interaction between DMMP and MOF-808 was clarified and found to be van der Waals interaction predominantly in Fig. 4g (blue denoted strong attractions including hydrogen bond, green indicated van der Waals interaction, and yellow represented strong repulsion including steric hindrance)^54^. The amorphous SiO_2_ has previously been regarded as a suitable adsorbent for chemical warfare agents due to the reliable affinity and stability of the resultant complex^55,56^. The well-orchestrated design of additional ceramic building blocks in MNAs may enable enhanced accumulation of DMMP on the porous channels. Simultaneously, the DMMP/SiO_2_ complex exhibited a similar van der Waals interaction which is weaker than that of DMMP/MOF-808 according to the value of simulated density gradient (Fig. 4h). Preferential DMMP adsorption at MOF-808 is also supported by a higher calculated binding enthalpy of −46.9 kJ mol^−1^ (compared to −45.9 kJ mol^−1^ at SiO_2_). In other words, the energy barrier based on van der Waals interactions facilitates the transfer of DMMP to adjacent MOF active sites in the interconnected channels for propelling the hydrolysis conversion of nerve agents, highlighting the advanced concept of the proposed structure of MNAs^57^.

## Discussion

We proposed that the combination of the freeze-drying method and interfacial engineering of silica sol templating allows the fabrication of superelastic MOF aerogels with hierarchical cellular architectures. To clarify the hydrogen bond-assisted interfacial coupling effect, the thorough evaluation of the contribution and mechanism of silica sol in MOF-808/SiO_2_ nanofibers aerogels was performed using MD and DFT simulations. The MOFs were well-anchored in the 3D interconnected nanofibrous networks and preserved their porosity, crystal structure, and accessible Lewis acid sites. Moreover, the ceramic-based channels generated a van der Waals barrier that facilitates preferential nerve agents accumulation in open MOF sites. With their properties of exceptional adsorption and catalytic decontamination performance towards DMMP, low filtration resistance, and elastic compressibility, we expect that these excellent MNAs will inspire the structural design of next-generation self-detoxifying PPE that could be deployed as outdoor emergency life-saving devices against CWAs threats either in liquid or in aerosol forms.

## Methods

### Preparation of electrospun SiO_2_ nanofibers

SiO_2_ nanofibers were prepared through a sol-gel electrospinning method^18^. Silica precursor sol solution was prepared by stirring dissolving tetraethyl orthosilicate (Shanghai energy chemical Co. Ltd., China), deionized water, and phosphoric acid (Aladdin, China) with a molar ratio of 1:10:0.01 for 12 h. 10 wt% poly(vinyl alcohol) (PVA, Mn = 86,000, Aladdin, China) aqueous solution was stirred at 80 °C for 6 h. Then, the silica sol was added to the PVA solution with a weight ratio of 1:1 and stirred for another 4 h. Following the electrospinning process was performed with a voltage of 20 kV and a constant feed rate of 1 mL h^−1^. Finally, the composite nanofibrous membranes were calcined at 800 °C in the air to obtain pure SiO_2_ nanofibrous membrane.

### Preparation of MNAs

In a standard process for preparing MNAs with a density of 12 mg cm^−3^, 0.4 g of Trimethoxymethylsilane (MTMS, Shanghai energy chemical Co. Ltd., China), and 0.003 g of oxalic acid (Aladdin, China) were firstly dissolved into a 100 g mixed solution (tert-butanol (Aladdin, China)/water, 1:4 by weight) and stirred for 30 min to form a silica sol. 0.6 g of SiO_2_ nanofibrous membranes were added to the silica sol and then homogenized for 5 min at 15,000 rpm using a high-speed disperser. Following that, 0.5 g of MOF-808 nanoparticles (Xian Qiyue Biology Co. Ltd., China) were added to the nanofibrous dispersions and stirred for 30 min. Finally, the resultant dispersions were poured into a cylindrical mold, quickly frozen in liquid nitrogen, and then freeze-dried for 1 day to produce the MNAs.

## Supplementary information

supporting information

## Data Availability

Source data are provided with this paper.

## Source data

Source Data
