# Supplementary material for: Interfacial engineered superelastic metal-organic framework aerogels with van-der-Waals barrier channels for nerve agents decomposition
Source: Nat Commun. 2023 Apr 13;14:2116. doi: 10.1038/s41467-023-37693-5 (PMC10101950; doi:10.1038/s41467-023-37693-5)
Supplement: Supplementary file 1 — supporting information [file 41467_2023_37693_MOESM1_ESM.pdf]

**Supplementary Information**

# Interfacial Engineered Superelastic Metal-Organic Framework Aerogels with Van-der-Waals Barrier Channels for Nerve Agents Decomposition

Yan *et al*.

**Supplementary Information contains:**

Supplementary Figures 1-21

Supplementary Tables 1-3

Supplementary Notes

Supplementary Discussion

Supplementary References

## Supplementary Figures

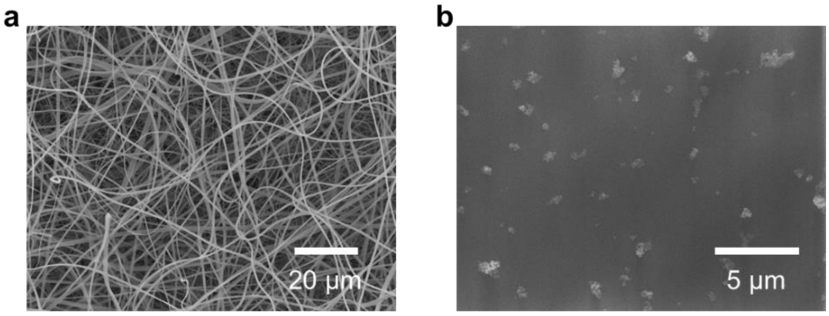


**Supplementary Figure 1.** SEM images of (a) $SiO_2$ nanofibers and (b) MOF-808 nanoparticles.

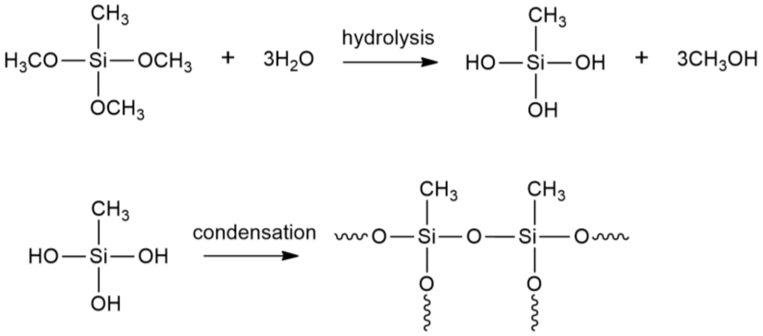


**Supplementary Figure 2.** The hydrolysis and condensation reactions of MTMS.[1]

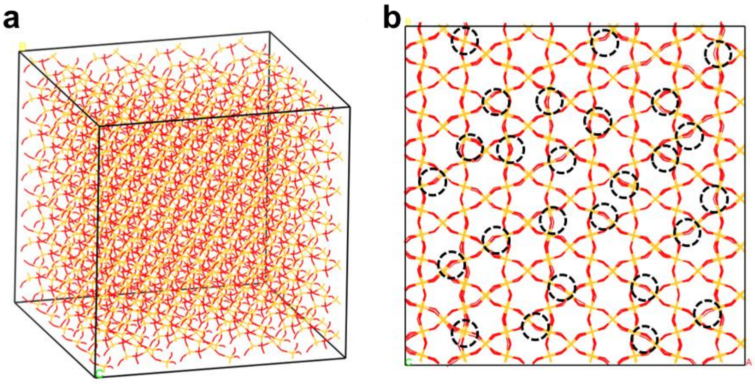


**Supplementary Figure 3.** A 3D view (a) and detailed front elevation (b) of an optimized amorphous $SiO_2$ cell. After the geometry optimization of the quartz cell, the cell was equilibrated and carried out by 500 ps of NVT ensemble dynamics at a cycling

temperature of 298 K-2000 K-298K for three times.

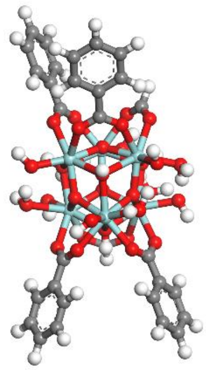

**Supplementary Figure 4.** The calculated MOF-808 model. Color code: red for oxygen, gray for carbon, white for hydrogen, and blue for zirconium.

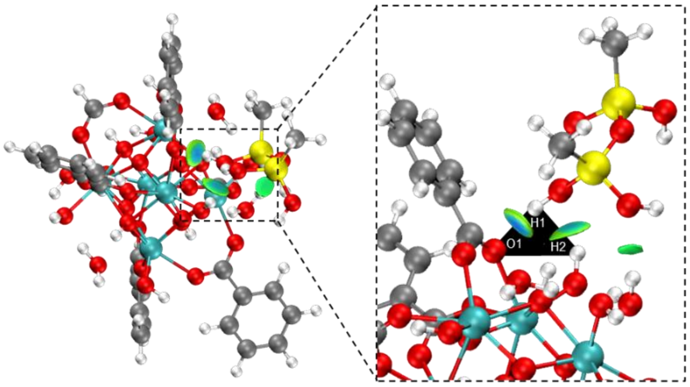


**Supplementary Figure 5.** The calculated silica sol cluster complex of MOF-808 model. The defined plane in Fig. 1f, 1g, and Supplementary Figure 6 is determined by O1, H2 atoms of MOF, and H1 atom of silica sol.

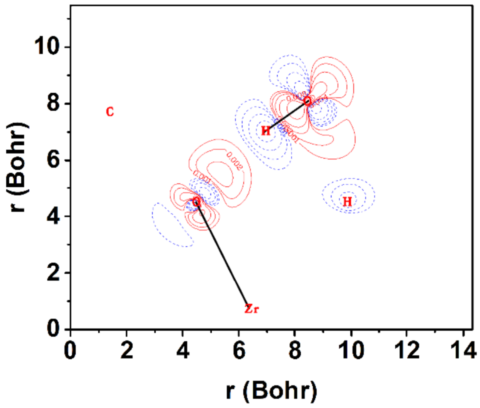


**Supplementary Figure 6.** Electron density difference map in the defined plane with detailed isovalues.

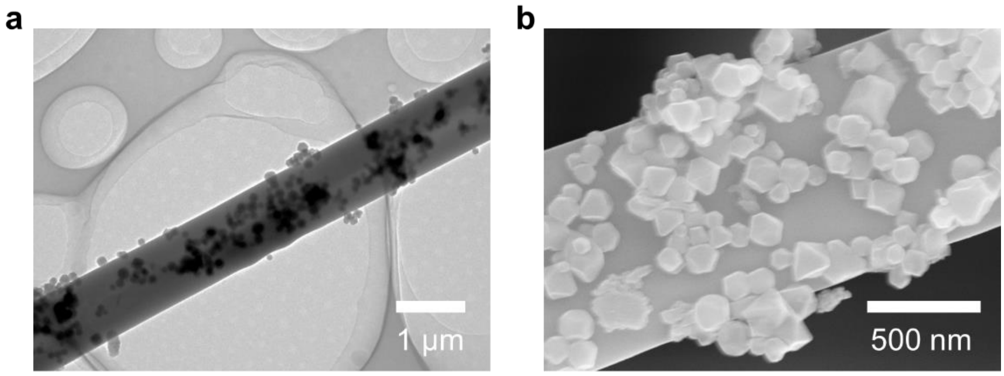


**Supplementary Figure 7.** (a) TEM and (b) high-mag SEM images of MNAs.

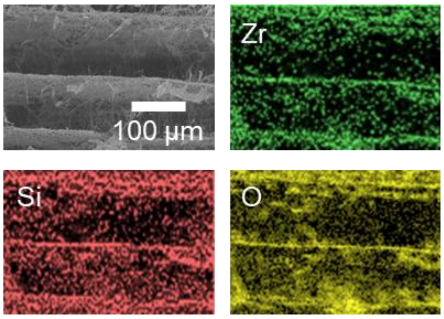


**Supplementary Figure 8.** EDX images of the MNAs.

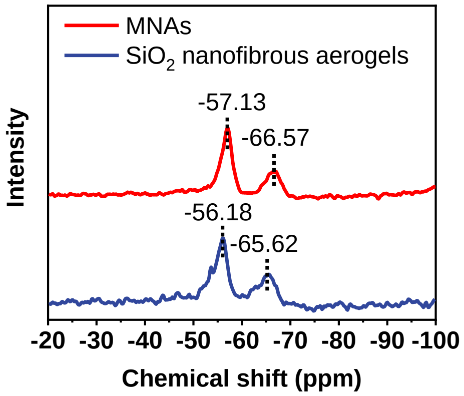


**Supplementary Figure 9.** $^{29}Si$ CP/MAS NMR spectra of MNAs and $SiO_2$ nanofibrous aerogels.

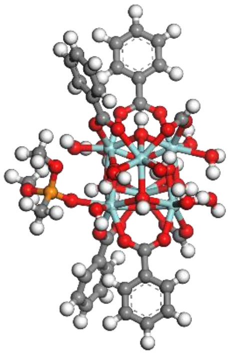

**Supplementary Figure 10.** The optimized MOF-808/DMMP complex. The nerve agent is bound to the MOF through the O (*sp*$^2$) Atom.

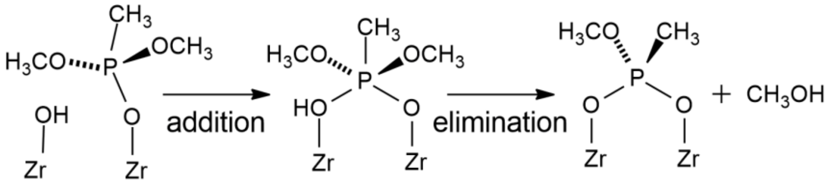


**Supplementary Figure 11.** The sketch of the essential steps in the reaction mechanism for degradation of DMMP on MOF-808.

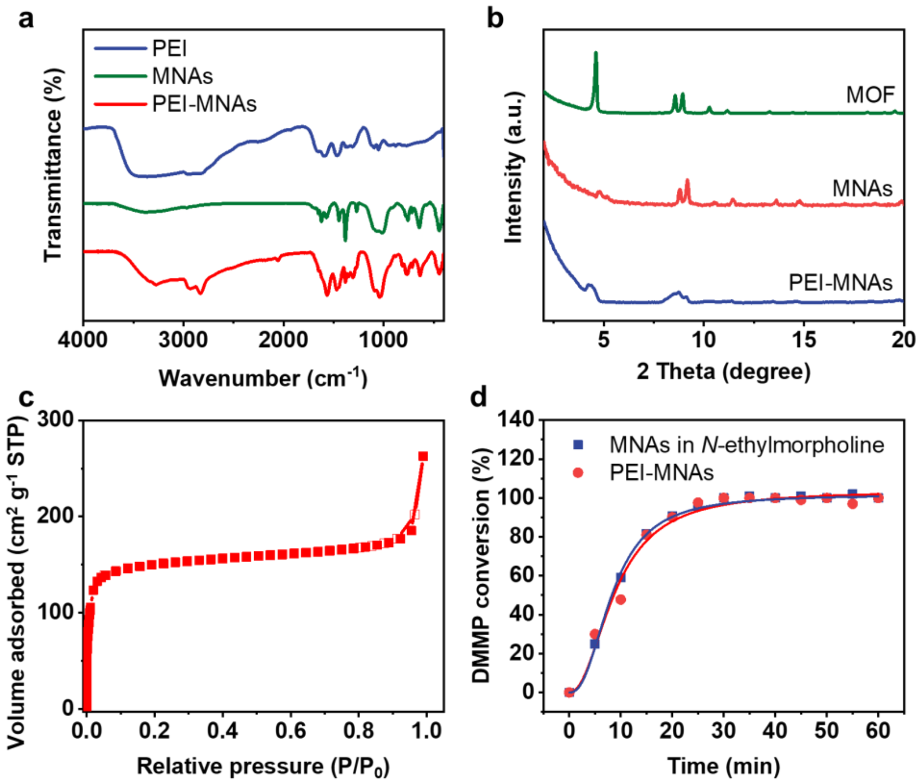


**Supplementary Figure 12.** (a) FT-IR, (b) XRD, and (c) Nitrogen adsorption-desorption isotherms of PEI-MNAs. (d) Comparison of hydrolysis profile of DMMP.

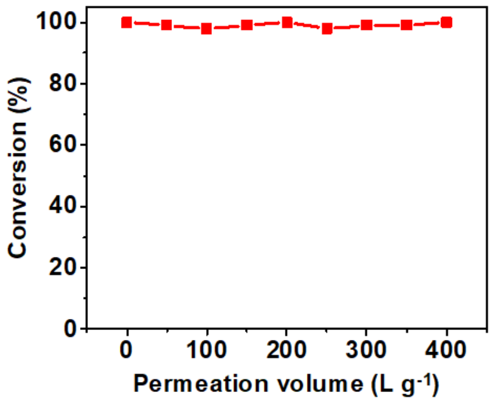


**Supplementary Figure 13.** The dynamic decontamination of the MNAs at a DMMP-contaminated flow (flow rate: 30 mL $h^{-1}$, DMMP concentration: 1 ppm).

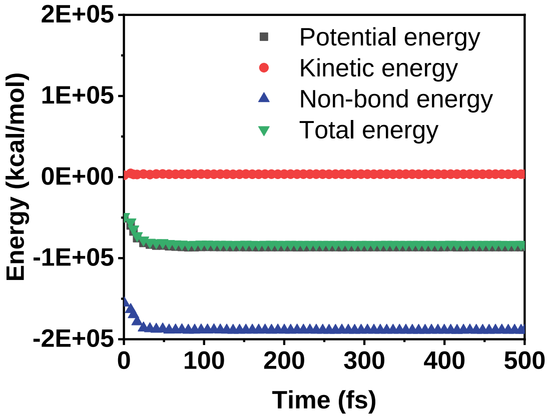


**Supplementary Figure 14.** Energy fluctuations versus time during the NVT ensemble for the amorphous $SiO_2$ cell. The energies were observed to fluctuate about a well-defined mean over the time scale of the dynamics, indicating that the equilibrium energy for the given condition had been attained and the system is in the most probable configuration.

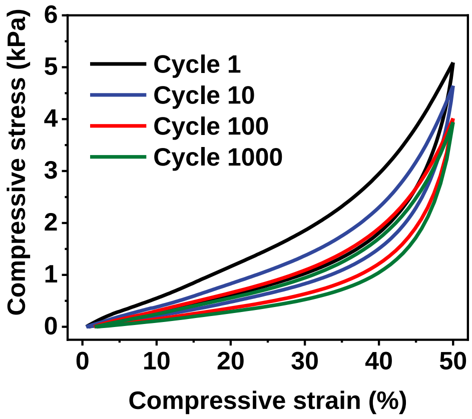


**Supplementary Figure 15.** The 1000 cyclic compressive $\sigma$ versus $\varepsilon$ curves of pure $SiO_2$ nanofibrous aerogels.

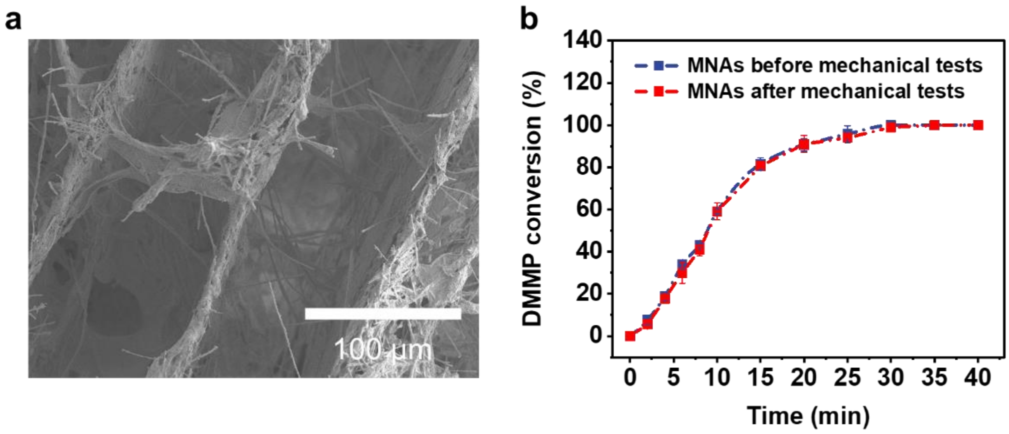


**Supplementary Figure 16.** (a) SEM image of MNAs after performing mechanical tests. (b) Comparison of the DMMP hydrolysis curves of MNAs before and after the mechanical tests.

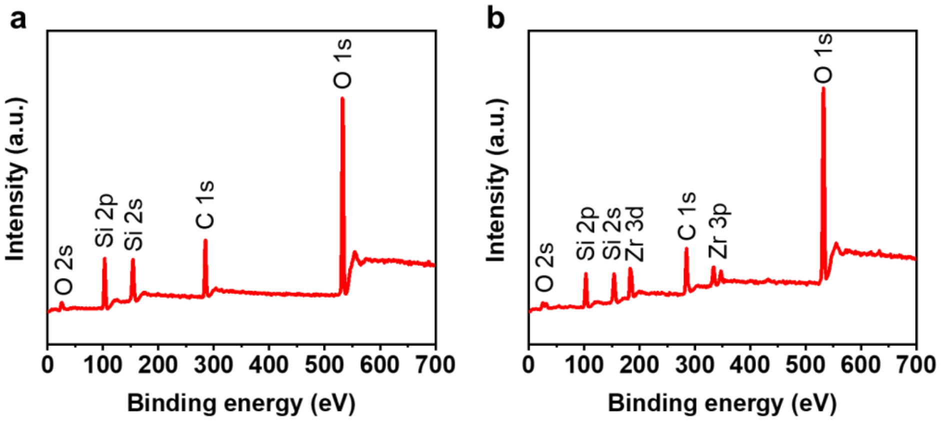


**Supplementary Figure 17.** XPS spectra of (a) $SiO_2$ nanofibrous aerogels and (b) MNAs for all elements.

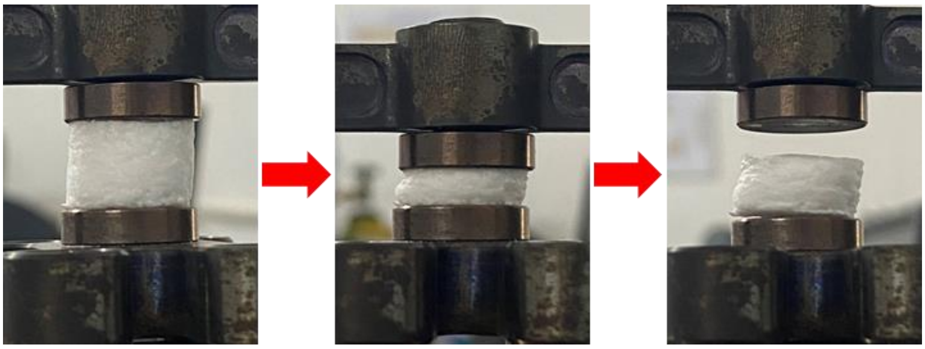

**Supplementary Figure 18.** Compressive properties of the composite aerogels (weight ratio of MOF-808 and $SiO_2$ nanofibers was 1:1).

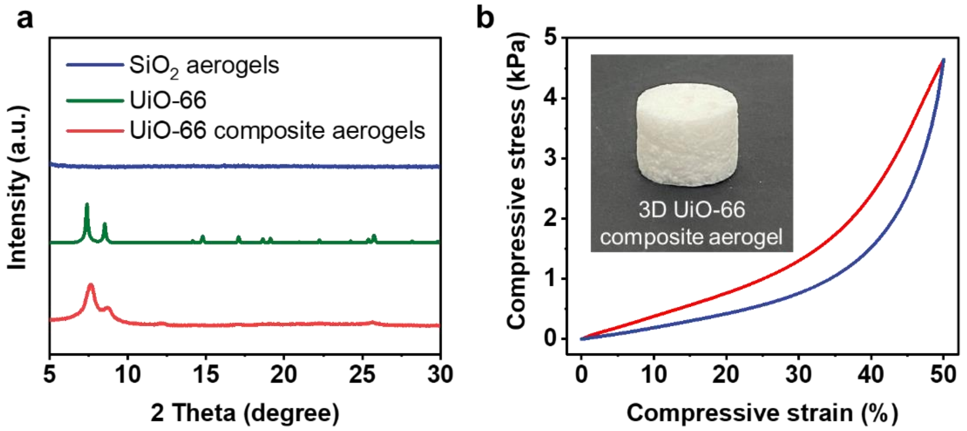


**Supplementary Figure 19.** Characterization of UiO-66 composite aerogels. (a) XRD, (b) compressive stress-strain curves. The insets show the photographs of the aerogel.

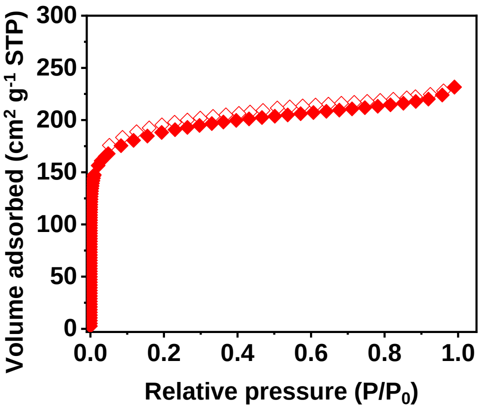


**Supplementary Figure 20.** Nitrogen adsorption-desorption isotherms of the mixture of MOF-808 nanoparticles and $SiO_2$ nanofibrous aerogels. The BET surface area of the mixture was estimated to be 681 $m^2$ $g^{-1}$.

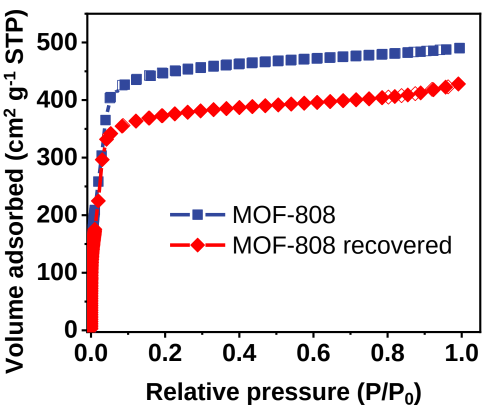


**Supplementary Figure 21.** Isotherms of MOF-808 nanoparticles and MOF-808

nanoparticles recovered from MNAs.

## Supplementary Tables

**Supplementary Table 1.** The characteristic peaks in the FT-IR spectra of the relative samples.

| Characteristic peak | $SiO_2$ aerogels | MOF-808 | MNAs |
|---|---|---|---|
| Si-O-Si | 1053 | / | 1014 |
| Si-O | 783 | / | 758 |
| | 451 | / | 443 |
| -OH | / | 3421 | 3373 |

**Supplementary Table 2.** The porous characteristics of $SiO_2$ nanofibrous aerogels, MOF-808, and MNAs.

| Sample | $S_{BET}$[a] ($m^2$/g) | $D_N$[b] | $D_S$[c] | $(D_S-D_N)/D_S\times100$ |
|---|---|---|---|---|
| $SiO_2$ aerogels | 5 | 2.53113 | 2.83018 | 10.5665 |
| MOF-808 | 1661 | 2.99533 | 2.99595 | 0.0207 |
| MNAs | 600 | 2.86502 | 2.93114 | 2.2558 |

[a]$S_{BET}$ was calculated by the BET method. [b]$D_N$ indicated the surface fractal dimension determined from the $N_2$ adsorption analysis method. [c]$D_S$ was the surface fractal dimension calculated from SAXS measurements.

**Supplementary Table 3.** The permeabilities of the 2D fibrous membranes and 3D aerogels.

| Permeability | MOF nanofibrous membranes | MOF aerogels (this work) |
|---|---|---|
| Pressure drop (Air) | 680.5 Pa | 220 Pa |
| Flux (Water) | 3900 L $m^{-2}$ $h^{-1}$ | 8100 L $m^{-2}$ $h^{-1}$ |

## Supplementary Notes

**Supplementary Note 1:**

*Characterization.* The morphology was measured by SEM (Tescan-Vega 3). The chemical structures were determined by XPS (Escalab 250Xi), EDS (JEM 2100F), FTIR (Nicolet iS10), and XRD (TD-3500). The porous structures were characterized by Brunauer-Emmett-Teller analyzer (ASAP 2020) and SAXS (SAXSess mc$^2$). The mechanical features were investigated by DMA (TA-Q850). The performance of air permeation was identified by filter tester (LZC-K). The detoxification was tracked by GC-MS (GC8890-5977B).

**Supplementary Note 2:**

*Decontamination Test:* DMMP (Sigma-Aldrich, China) decomposition was measured using a modified version of a previously described procedure.[2] The DMMP solution was prepared by mixing 2.35 mL of *N*-ethylmorpholine and 4 μL of DMMP into 35 mL of deionized water. 200 mg of MNAs were carefully added to 3.5 mL of the aforementioned DMMP solution. Periodic monitoring of the decomposition was carried out by removing a 10 μl aliquot from the reaction mixture. In addition, the extraction of the decomposition product MMPA was achieved by stirring the samples in an eluent of 0.05% formic acid (pH = 2.32) for 24 h at 60 ℃.[3]

**Supplementary Note 3:**

*Small-angle x-ray scattering (SAXS)*: SAXS was conducted using Beamline BL16B1 of the Shanghai Synchrotron Radiation Facility (SSRF). The beam with 1.2 × 0.12 mrad$^2$ divergence was accepted by the optics system. Generally, the beamline worked at 10 keV (0.124 nm) for experiments. The distance of the sample to the SAXS

detector was adjusted up to 5 m. The detectors were Mar165 CCD for SAXS. Silver behenate was used to calibrate the scattering vector (q). Fit2D software was used to analyze the 2D SAXS data.

**Supplementary Note 4:**

*Molecular computational details:*

MD: An equilibrated (298 K) quartz cell with a cubic edge length of 3.5 nm was heated to 2000 K in the NVT ensemble, resulting in the melting of the solid. After the equilibration of 2000 K, it was cooled to 298K and equilibrated. The alternate circle of heating and cooling was repeated 3 times. The final structure obtained is the equilibrium configuration of the amorphous silica (Supplementary Fig. 3). The 3D lattice of the silica sol was constructed by Amorphous cell suite. The optimization in the NVT ensemble was performed at 298 K under the COMPASS force field. The silica sol was completely relaxed to ensure that the cell parameters and energies remained constant during the MD simulations. The crystallographic data of MOF-808 was obtained by CCDC 1944650.

DFT: The geometries were optimized at the DFT-B3LYP/6-31+G(d,p) level of theory. The mechanistic studies have been carried out using a cluster model of MOF-808, formed by extracting one zirconium node from a periodically optimized structure. There are 6 linkers (four benzoate and two formate groups). The para-carbon atoms of the benzoate groups have been frozen to mimic the rigidity of the periodic MOF structure (Supplementary Fig. 4).

**Supplementary Note 5: XYZ coordinates**

*The MOF/silica sol complex in Fig. 1*

| | | | |
|---|---|---|---|
| Zr | 0.66576900 | -1.28574200 | -2.27081700 |

| | | | |
|---|---|---|---|
| Zr | -0.55553600 | -2.93968300 | 0.66470300 |
| Zr | 1.25542400 | 0.05236300 | 0.91713500 |
| Zr | -2.26499100 | 0.10003300 | 1.69417700 |
| Zr | -1.03822100 | 1.72688600 | -1.22971700 |
| Zr | -2.85681600 | -1.30800200 | -1.53386100 |
| C | 3.63743700 | -0.77027900 | -1.13471600 |
| C | 5.08081500 | -1.09185400 | -1.30115000 |
| C | 5.66369100 | -3.30121800 | 3.90175300 |
| C | 5.96912100 | -0.88211100 | -0.23887600 |
| C | 4.58362100 | -2.61680100 | 3.35149600 |
| C | 1.44213400 | -4.35451500 | -1.39626100 |
| C | 6.90161400 | -1.91995800 | -2.65766800 |
| C | 4.47328900 | -5.39080200 | 3.64611200 |
| C | 2.28195100 | -2.59121900 | 2.34841000 |
| C | 7.31688900 | -1.19160300 | -0.38611100 |
| C | 3.43874400 | -3.31615100 | 2.94864700 |
| C | 5.55201100 | -1.61618000 | -2.51067700 |
| C | 5.60854000 | -4.68798500 | 4.05210000 |
| C | 7.78184900 | -1.70726800 | -1.59627100 |
| C | 3.39273500 | -4.70848400 | 3.09517300 |
| C | -5.22672800 | -0.55333800 | 0.58634200 |
| C | -6.68056300 | -0.30801000 | 0.79204500 |
| C | -5.70612600 | 5.31875900 | 2.39311900 |
| C | -7.59937400 | -0.59579000 | -0.22430700 |
| C | -5.72149800 | 6.41765700 | 1.53338300 |

| | | | |
|---|---|---|---|
| C | -2.96623100 | 3.21266400 | 0.85521300 |
| C | -8.47192400 | 0.56889800 | 2.16107600 |
| C | -3.89179600 | 4.35083100 | 1.11671400 |
| C | -3.64399000 | 1.32718900 | -3.22605300 |
| C | -8.94643200 | -0.29327500 | -0.05051900 |
| C | -4.81871000 | 6.49034300 | 0.47148200 |
| C | -7.12485200 | 0.27150100 | 1.98769700 |
| C | -4.79250700 | 4.28961300 | 2.18788200 |
| C | -9.38022000 | 0.29286300 | 1.13907800 |
| C | -3.90872500 | 5.45909000 | 0.26047900 |
| H | -3.68840400 | 1.17107000 | -0.55642200 |
| H | 1.80230400 | 1.26054700 | -1.60250100 |
| H | -1.14796000 | -3.54664700 | -2.07112000 |
| H | -0.12222100 | -1.22307800 | 3.05874300 |
| H | -2.05238500 | 1.58921900 | 3.81246300 |
| H | -2.37701400 | -2.53559100 | 2.97105800 |
| H | -2.68020300 | -1.31303100 | 3.97620300 |
| H | -4.62031300 | -3.23304000 | -1.36298400 |
| H | -1.61037200 | -2.04633400 | -4.04805100 |
| H | -2.94553200 | -2.89276600 | -3.77642300 |
| H | -0.69713700 | 2.93353400 | -3.52112600 |
| H | 0.99521500 | 3.65583600 | -1.04764000 |
| H | 1.04958900 | 2.96656500 | 0.38808300 |
| H | 2.92475000 | 2.16929100 | 1.49320800 |
| H | -0.72325900 | -4.00610600 | 3.08840500 |

| | | | |
|---|---|---|---|
| H | -2.85101800 | -4.11821700 | -0.57734400 |
| H | -2.56298700 | -4.73278900 | 0.89727900 |
| H | 0.40268800 | -2.91036600 | -4.36247400 |
| H | 0.64291500 | 1.25077100 | -3.63208500 |
| H | 0.64056300 | -0.00580800 | -4.65165200 |
| H | 5.58424000 | -0.48383900 | 0.69296900 |
| H | 4.60732500 | -1.53952800 | 3.23032100 |
| H | 4.84925100 | -1.77565100 | -3.32088700 |
| H | 6.45187700 | -5.22088000 | 4.48257300 |
| H | 8.83543400 | -1.94708200 | -1.71211100 |
| H | 2.50595500 | -5.23986100 | 2.76779500 |
| H | -7.24092400 | -1.03976000 | -1.14667400 |
| H | -6.43650300 | 7.22030100 | 1.69234700 |
| H | -6.39912800 | 0.48442100 | 2.76441600 |
| H | -4.77043700 | 3.42384300 | 2.84034000 |
| H | -10.43211600 | 0.53181800 | 1.27086400 |
| H | -3.20704200 | 5.49262800 | -0.56567400 |
| O | 2.85828800 | -1.06543200 | -2.09970700 |
| O | 1.08201200 | 0.37667600 | -3.87753300 |
| O | -4.86088000 | -1.13348600 | -0.48730200 |
| O | -3.70243700 | -3.25124800 | -1.66820100 |
| O | -1.11486900 | -0.21757500 | -2.06858900 |
| O | 0.80167500 | -1.60850400 | -0.21122700 |
| O | 0.69757600 | -4.44613900 | -0.37995000 |
| O | -2.15920000 | -4.52113100 | 0.04152300 |

| | | | |
|---|---|---|---|
| O | 2.36205200 | -1.31518600 | 2.25999800 |
| O | -2.69979700 | 2.05872900 | -2.81152000 |
| O | -0.05336600 | 2.56941200 | -2.89714500 |
| O | -2.92870300 | 2.27889400 | 1.71299600 |
| O | -1.98474000 | 0.64095500 | 3.64300300 |
| O | -2.13653000 | -1.62526900 | 0.41442900 |
| O | -0.60055600 | 0.96614200 | 0.68845100 |
| O | -4.44897500 | -0.12903900 | 1.49473900 |
| O | -2.90884200 | -1.70088900 | 3.11738400 |
| O | 3.27786600 | -0.21048600 | -0.05297600 |
| O | 2.00827500 | 1.85674000 | 1.49495000 |
| O | -0.31475000 | -1.04139500 | 2.13092600 |
| O | -2.87044100 | 0.67294900 | -0.45516600 |
| O | -2.29854200 | 3.23978500 | -0.23229500 |
| O | 0.45277100 | 3.36222000 | -0.30239700 |
| O | -3.94929700 | 0.16180500 | -2.84717300 |
| O | -2.58276100 | -1.99494900 | -3.79979900 |
| O | 1.28351200 | -3.28154000 | 1.98959300 |
| O | -1.37906100 | -3.75956500 | 2.42375100 |
| O | 1.50250700 | -3.42569400 | -2.24737200 |
| O | 0.05964000 | -2.03136300 | -4.15541100 |
| O | 1.05395000 | 0.78627200 | -1.22098300 |
| O | -1.05349500 | -2.71134300 | -1.59889700 |
| H | 4.43140800 | -6.47024100 | 3.75838400 |
| H | 6.54836900 | -2.75463600 | 4.21526900 |

| | | | |
|---|---|---|---|
| H | 8.00509700 | -1.03246800 | 0.43886200 |
| H | 7.26822700 | -2.32284900 | -3.59720000 |
| H | -4.82853900 | 7.34873800 | -0.19386500 |
| H | -6.40802400 | 5.26363400 | 3.22008600 |
| H | -9.65760700 | -0.50869500 | -0.84259400 |
| H | -8.81417500 | 1.01866500 | 3.08847300 |
| H | -4.28875200 | 1.76323600 | -4.00754700 |
| H | 2.10836900 | -5.21583400 | -1.57475200 |
| Si | 4.76442800 | 3.38136200 | -0.72548700 |
| O | 3.28312100 | 4.11459600 | -0.93238800 |
| H | 3.11963300 | 4.86523600 | -0.34793400 |
| O | 5.81890600 | 4.48208100 | -0.12410300 |
| O | 4.68803300 | 2.20067700 | 0.46647700 |
| H | 4.30323100 | 1.33036000 | 0.23954200 |
| C | 5.25791600 | 2.67461400 | -2.37187900 |
| H | 4.47900200 | 2.02698700 | -2.78821600 |
| H | 5.42733700 | 3.48484000 | -3.08755700 |
| H | 6.17686300 | 2.08655400 | -2.29247100 |
| Si | 6.99396400 | 4.57035900 | 1.06516800 |
| O | 6.64781100 | 5.82389900 | 2.08911800 |
| H | 6.10154000 | 5.59861600 | 2.85000500 |
| O | 6.98120000 | 3.13481600 | 1.88451900 |
| H | 6.25130200 | 2.55029100 | 1.61813400 |
| C | 8.62997400 | 4.94561300 | 0.27196800 |
| H | 8.86866800 | 4.21356400 | -0.50535600 |

| | | | |
|---|---|---|---|
| H | 8.61463300 | 5.93887200 | -0.18705800 |
| H | 9.43210700 | 4.92922700 | 1.01572300 |

*The MOF/DMMP complex in Fig. 4*

| | | | |
|---|---|---|---|
| Zr | 1.59288300 | -2.18148000 | -1.40604900 |
| Zr | 1.11818600 | -1.37303800 | 2.02792500 |
| Zr | 1.56895000 | 1.23557700 | -0.36930800 |
| Zr | -1.66056400 | 0.74254500 | 1.15857400 |
| Zr | -1.16802700 | -0.06616700 | -2.28002000 |
| Zr | -1.63161500 | -2.65404200 | 0.10774600 |
| C | 4.11539300 | -0.25714700 | -1.85063700 |
| C | 5.56952300 | -0.18593700 | -2.15868500 |
| C | 6.94306900 | 2.42039700 | 2.70051200 |
| C | 6.29577600 | 0.96660900 | -1.83355800 |
| C | 5.68415800 | 2.14748600 | 2.17284800 |
| C | 3.51558800 | -3.27546800 | 1.02415100 |
| C | 7.57827200 | -1.20080500 | -3.03968600 |
| C | 6.67626600 | 0.60932300 | 4.28089100 |
| C | 3.56600600 | 0.79961600 | 2.13277400 |
| C | 7.65716900 | 1.03332000 | -2.10942400 |
| C | 4.91117900 | 1.10496300 | 2.69981600 |
| C | 6.21745200 | -1.27174600 | -2.75959600 |
| C | 7.43901900 | 1.65370600 | 3.75644100 |
| C | 8.29595300 | -0.04927300 | -2.71484400 |
| C | 5.41777000 | 0.33444000 | 3.75428800 |
| C | -4.12516800 | -1.27357900 | 1.70487200 |

| | | | |
|---|---|---|---|
| C | -5.55911300 | -1.36612300 | 2.09414900 |
| C | -6.89636500 | 3.57300700 | -0.90423200 |
| C | -6.28288600 | -2.54176800 | 1.86168200 |
| C | -7.33874100 | 3.62055700 | -2.22673100 |
| C | -3.53729600 | 1.88089100 | -1.30655400 |
| C | -7.56017500 | -0.30371200 | 2.94220600 |
| C | -4.83887200 | 2.52830000 | -1.63442500 |
| C | -3.38853200 | -2.47306300 | -2.60727700 |
| C | -7.64104400 | -2.59312700 | 2.16009500 |
| C | -6.53102600 | 3.13188500 | -3.25470900 |
| C | -6.20402300 | -0.24887400 | 2.64061800 |
| C | -5.64926900 | 3.03146700 | -0.60851400 |
| C | -8.27888200 | -1.47333400 | 2.69497600 |
| C | -5.28640000 | 2.58381000 | -2.96044000 |
| H | -3.38554300 | -0.61652900 | -0.67497700 |
| H | 1.58677800 | 0.13261200 | -3.04435000 |
| H | 0.85569400 | -3.92887200 | 0.77270100 |
| H | 0.77117500 | 1.57838500 | 2.30571400 |
| H | -2.13900200 | 3.23025200 | 1.47242400 |
| H | -0.75471900 | 0.00995500 | 3.84241300 |
| H | -1.56567300 | 1.40450400 | 3.81030400 |
| H | -2.47280800 | -4.18853800 | 2.05041400 |
| H | -0.15876500 | -4.60516400 | -1.43281000 |
| H | -1.04817900 | -5.29731500 | -0.28419200 |
| H | -1.33401100 | -1.03353400 | -4.70694000 |

| | | | |
|---|---|---|---|
| H | -0.21802100 | 1.56071100 | -4.21238600 |
| H | 0.34052400 | 2.29189200 | -2.89207000 |
| H | 2.45654900 | 2.48208900 | -2.50413400 |
| H | 1.32436700 | -0.31506600 | 4.44421300 |
| H | -0.49084400 | -3.66801900 | 2.66381900 |
| H | 0.01651700 | -2.86649000 | 3.97866800 |
| H | 2.01289100 | -4.80653900 | -1.62967300 |
| H | 0.58464000 | -1.71085400 | -4.05129100 |
| H | 1.11968300 | -3.22511900 | -3.82882900 |
| H | 5.77798700 | 1.79235800 | -1.35894400 |
| H | 5.28123600 | 2.73085600 | 1.35237000 |
| H | 5.63959400 | -2.15735000 | -2.99957700 |
| H | 8.42186100 | 1.86779800 | 4.16747500 |
| H | 9.35956100 | 0.00337400 | -2.93194600 |
| H | 4.81372000 | -0.47740700 | 4.14413900 |
| H | -5.77125800 | -3.39713700 | 1.43396800 |
| H | -8.31450700 | 4.03966000 | -2.45716100 |
| H | -5.62545600 | 0.65106100 | 2.81590800 |
| H | -5.29240900 | 2.97339200 | 0.41392400 |
| H | -9.34054100 | -1.51472100 | 2.92365100 |
| H | -4.64973900 | 2.18573700 | -3.74293300 |
| O | 3.52831600 | -1.36537100 | -2.06907100 |
| O | 1.35493400 | -2.28719700 | -3.74383600 |
| O | -3.55385600 | -2.32528700 | 1.26867100 |
| O | -1.62637700 | -4.18180700 | 1.58220800 |

| | | | |
|---|---|---|---|
| O | -0.47686600 | -1.90368200 | -1.49942600 |
| O | 1.79152300 | -0.78168700 | 0.13817200 |
| O | 2.89163900 | -2.73410200 | 1.97936700 |
| O | 0.30314000 | -3.27527800 | 3.14674400 |
| O | 3.18369400 | 1.49443400 | 1.14100500 |
| O | 1.21978000 | 3.30373600 | 0.64534300 |
| O | -2.83264100 | -1.44585500 | -3.09016300 |
| O | -0.59780900 | -0.58498600 | -4.26796400 |
| O | -3.14512100 | 1.94426500 | -0.10263300 |
| O | -1.71957100 | 2.59708600 | 2.07065300 |
| O | -0.87645800 | -1.22003300 | 1.43433500 |
| O | -0.48865300 | 1.03595300 | -0.60809400 |
| O | -3.59065500 | -0.12799700 | 1.80051900 |
| O | -1.58758500 | 0.47708000 | 3.53293500 |
| O | 3.55646400 | 0.79725900 | -1.41946400 |
| O | 1.64723800 | 2.63237900 | -1.99722800 |
| O | 0.54067600 | 0.87389000 | 1.68841400 |
| O | -2.43747100 | -0.63192800 | -0.50779700 |
| O | -2.93354300 | 1.28265900 | -2.25478600 |
| O | -0.48375300 | 1.92991700 | -3.35583000 |
| O | -3.20471800 | -3.00355600 | -1.47697300 |
| O | -1.07774600 | -4.66723400 | -1.01932100 |
| O | 2.89704100 | -0.12664900 | 2.68609400 |
| O | 0.63878100 | -0.85339800 | 4.02840000 |
| O | 3.21408400 | -3.28831500 | -0.20022700 |

| | | | |
|---|---|---|---|
| O | 1.33653800 | -4.21869300 | -1.99029400 |
| O | 1.10965300 | -0.08638400 | -2.23593800 |
| O | 0.59419000 | -3.04158100 | 0.50132800 |
| H | 7.06454400 | 0.00832500 | 5.09813700 |
| H | 7.53891400 | 3.23085500 | 2.29028300 |
| H | 8.22171700 | 1.92510400 | -1.85289100 |
| H | 8.08062200 | -2.04139000 | -3.50946400 |
| H | -6.87581500 | 3.17228900 | -4.28392900 |
| H | -7.52667300 | 3.95197700 | -0.10487600 |
| H | -8.20425400 | -3.50249600 | 1.97158600 |
| H | -8.05863800 | 0.56350500 | 3.36555700 |
| H | -4.14064600 | -2.96195900 | -3.24997800 |
| H | 4.43348800 | -3.82121600 | 1.30273200 |
| C | 1.66275300 | 5.58527500 | -0.92742200 |
| H | 2.65523200 | 5.74586300 | -0.50036100 |
| H | 1.74621400 | 4.96194000 | -1.82000400 |
| H | 1.20831600 | 6.55250200 | -1.15332300 |
| P | 0.67667000 | 4.65208800 | 0.25417000 |
| O | -0.81076700 | 4.47850300 | -0.33282100 |
| O | 0.58220000 | 5.66215900 | 1.50065400 |
| C | 0.25782200 | 5.17346700 | 2.83016600 |
| H | 1.17741700 | 4.84112200 | 3.31863800 |
| H | -0.15807700 | 6.02523900 | 3.37014200 |
| H | -0.46324200 | 4.35217300 | 2.78697700 |
| C | -1.60863400 | 5.58950300 | -0.77726000 |

| | | | |
|---|---|---|---|
| H | -1.22851800 | 5.98220300 | -1.72523800 |
| H | -2.61419200 | 5.19695400 | -0.92995400 |
| H | -1.63108900 | 6.38311400 | -0.02389300 |

*The silica/DMMP complex in Fig. 4*

| | | | |
|---|---|---|---|
| Si | 0.13864900 | -0.78589500 | 0.07316800 |
| O | -0.85693300 | -2.05581600 | 0.01120200 |
| H | -1.72562900 | -1.90952300 | -0.43471800 |
| O | -0.68185500 | 0.60361000 | 0.43241900 |
| O | 0.95178700 | -0.51858300 | -1.34225300 |
| O | 1.24917200 | -1.05903000 | 1.25924400 |
| Si | -0.60363500 | 2.19534900 | -0.00154400 |
| H | -1.50686600 | 2.94893500 | 0.89556300 |
| H | -0.97742800 | 2.37514400 | -1.42148400 |
| O | 0.93386100 | 2.77960700 | 0.21739200 |
| Si | 2.48483900 | 2.23219500 | 0.17565600 |
| H | 3.39321400 | 3.37458500 | 0.29909400 |
| O | 2.72942300 | 1.18474300 | 1.42706900 |
| Si | 2.65500400 | -0.41675800 | 1.82379200 |
| H | 2.73176700 | -0.55497400 | 3.28006300 |
| Si | 2.40152100 | -0.01445900 | -1.93429900 |
| H | 2.32637000 | 0.12556500 | -3.39001600 |
| O | 2.76638700 | 1.44760400 | -1.25036000 |
| O | 3.92460400 | -1.20774600 | 1.13972000 |
| O | 3.58113700 | -1.09409800 | -1.56147100 |
| Si | 4.39170900 | -1.83166200 | -0.32171300 |

| | | | |
|---|---|---|---|
| H | 5.83856200 | -1.57607900 | -0.48299600 |
| H | 4.08924900 | -3.27872800 | -0.35874900 |
| C | -4.89667000 | 1.93178400 | 0.01230900 |
| H | -5.13636500 | 1.69130800 | 1.05185100 |
| H | -4.32978700 | 2.86238600 | -0.02511100 |
| H | -5.82149800 | 2.05105400 | -0.56191800 |
| C | -3.48315400 | -1.02219700 | 2.09445900 |
| H | -3.90575400 | -1.20771500 | 3.08189300 |
| H | -2.88126700 | -0.11013000 | 2.10698100 |
| H | -2.85844800 | -1.86114700 | 1.78330700 |
| C | -6.01484700 | -0.97701100 | -1.04973400 |
| H | -6.25453800 | -2.03267900 | -0.90798500 |
| H | -6.00865100 | -0.75319100 | -2.11880000 |
| H | -6.77429800 | -0.37016900 | -0.55176500 |
| P | -4.36762200 | -0.65992000 | -0.38002100 |
| O | -3.27969700 | -1.47337100 | -0.99345700 |
| O | -4.06189900 | 0.91968900 | -0.57626300 |
| O | -4.61467400 | -0.87336900 | 1.19951500 |

## Supplementary Discussion

*Intermolecular potential in the confined shear simulation:* The chains consist of 20 segments which are connected in a freely joined topology. The segments of the same chain as well as segments belonging to different chains interact via a pairwise purely repulsive, shifted and truncated 12-6 Lennard-Jones (LJ) potential [4-6]:

$$U(\gamma)=\begin{cases} 4\varepsilon\left[\left(\frac{\sigma}{\gamma}\right)^{12}-\left(\frac{\sigma}{\gamma}\right)^{6}-\frac{1}{4}\right], & \gamma\leq\sqrt[6]{2}\sigma \\ 0, & \gamma>\sqrt[6]{2}\sigma \end{cases} \tag{1}$$

where $\gamma$ is the distance between the centers of the two segments, $\varepsilon$ is the LJ energy parameter and $\sigma$ is the LJ length parameter. For successive monomers of the chain a strongly attractive FENE (finite extensibility non elastic) spring potential is added:

$$U_{\mathrm{bond}}(\gamma)=-\frac{\kappa}{2}R_0^2\ \mathrm{In}\left[1-\left(\frac{\gamma}{R_0}\right)^2\right],\quad \gamma<R_0 \tag{2}$$

This model has been studied extensively for chains both in the bulk, confined between walls, and under shear. The interaction between walls and segments is modelled by a pairwise LJ potential which includes the attractive tail of the potential:

$$U_w(\gamma)=4\varepsilon_w\left[\left(\frac{\sigma_w}{\gamma}\right)^{12}-\left(\frac{\sigma_w}{\gamma}\right)^{6}\right] \tag{3}$$

*Fractal analysis:* The $N_2$ adsorption method was according to the modified Frenkel-Halsey-Hill (FHH) theory[7] of multilayer gas adsorption:

$$\mathrm{In}\left(\frac{v}{v_{mono}}\right)=A\left[\mathrm{In}\left(\mathrm{In}\frac{p_0}{p}\right)\right]+c \tag{4}$$

where $v$ is the amount of $N_2$ adsorbed at each equilibrium pressure, $v_{mono}$ is the amount adsorbed of monolayer coverage, $\rho_0$ is the saturation pressure.

Scattering intensity derived from SAXS[8]:

$$I(q) = 4\pi(\rho_1 - \rho_2)^2 \varphi(1-\varphi)V\int_0^{\infty} r^2\gamma_0(r)\frac{\sin(qr)}{qr} \tag{5}$$

where $I$(q) is the scattering intensity, $\rho_1$ and $\rho_2$ are the scattering length densities of the matrix and the pores, respectively, $\varphi$ is the volume fraction of pores, $V$ is the scattering volume, $\gamma$ is the radius of pores, $\theta$ is the scattering angle, $\lambda$ is the wavelength of X rays, $q$ is the scattering vector, $A$ is the diameter of pores. The scattering intensity follows the power law in the fractal system:

$$I(q) = I_0 q^{-\alpha} \tag{6}$$

*The performance comparison*: We compared the air pressure drop at a constant velocity and the water flux driven by gravity of the published MOF composite fabrics and MNAs. As shown in Supplementary Table 3, the obtained aerogels in our research possessed a lower diffusion resistance than that of traditional MOF composite fabrics (~68% reduction in air[9] and ~52% reduction in liquid[10]). In contrast, the superior spatial dispersion of the MNAs enabled a more accessible MOF catalyst in the hierarchical network for enhanced reagent diffusion and subsequently a faster reaction rate, highlighting the advanced concept of the proposed 3D structure of our MNAs, which is not easily obtainable by previously reported materials. In another comparison study, MNAs showed a higher liquid uptake of 8710 wt% than that of the published MOF composites (1650 wt%[11]), which was attributed to their lower density (12 mg $cm^{-3}$). The high liquid uptake and simultaneous rapid decomposition make this material a promising detoxifying adsorbent for nerve agents spill. Take the detoxification of

DMMP (a sarin surrogate) for example, the half-life of the MNAs was 5.29 min, which was much better than that of the published MOF composite fabrics (50 min[12]).

*Nonvolatile bases:* We propose a facile strategy to achieve the alkaline test condition by using the non-volatile alkali polymers such as polyethyleneimine (PEI) instead of the traditional aqueous solutions. We integrated the non-volatile alkali polymers into the hybrid aerogels by adding 0. 4 mmol PEI in the 100 g dispersion before freeze-drying. The obtained aerogels were denoted as PEI-MNAs. This is a significant step forward in the integration of MOFs and bases together into a porous monolith to access a catalytically active protective material, which is a popular solution for the destruction of these harmful chemicals in practical environments. Evidence of the formation of PEI-MNAs was obtained from FT-IR and XRD analysis (Supplementary Fig. 12). The specific surface area of PEI-MNAs was estimated to be 568 $m^2$ $g^{-1}$. Remarkably, the catalytic performances of PEI-MNAs are comparable to the MNAs using the volatile *N*-ethylmorpholine solution, highlighting their potential for incorporation into protective layers against CWAs in real situations. We believe the findings pave the way for the deployment of MOF materials in personal protective equipment.

## Supplementary References

1 Wang, F., Dou, L., Dai, J., Li, Y., Huang, L., Si, Y., Yu, J., Ding, B. In situ synthesis of biomimetic silica nanofibrous aerogels with temperature-invariant superelasticity over one million compressions. *Angew. Chem. Int. Ed.* **132**, 8362 (2020).

2 Moon, S., Wagner, W., Mondloch, E., Peterson, W., DeCoste, B., Hupp, T., Farha, K. Effective, Facile, and Selective Hydrolysis of the chemical warfare agent vx using $Zr_6$-based metal-organic frameworks. *Inorg. Chem.* **54**, 10829 (2015).

3 Goncharova, N., Statkus, A., Nesterenko, N., Tsysin, I., Zolotov, A. Solid-phase extraction of alkylphosphonic and O-alkyl alkylphosphonic acids followed by HPLC separation using porous graphitic carbon sorbent. *J. Chromatogr. A* **1653**, 462420 (2021).

4 Manias, E., Hadziioannou, G., Bitsanis, I., Brinke, G. Stick and slip behavior of confined oligomer melts under shear. A molecular-dynamics study. *Europhys. Lett.* **24**, 99 (1993).

5 Tamura, H., Yoshida, M., Kusakabe, K., Mo, C., Miura, R., Kubo, M., Teraishi, K., Chatterjee, A., Miyamoto, A. Molecular dynamics simulation of friction of hydrocarbon thin films. *Langmuir* **15**, 7816 (1999).

6 Jiang, C., Ouyang, J., Li, W., Wang, X., Wang, L. The effects of wall roughness on the methane flow in nano-channels using non-equilibrium multiscale molecular dynamics simulation. *Microfluid. Nanofluid.* **21**, 92 (2017).

7 Si, Y., Ren, T., Ding, B., Yu, J., Sun, G. Synthesis of mesoporous magnetic Fe3O4@carbon nanofibers utilizing in situ polymerized polybenzoxazine for water purification. *J. Mater. Chem.* **22**, 4619 (2012).

8 Radlinski, P., Mastalerz, M., Hinde, L., Hainbuchner, M., Rauch, H., Baron, M., Lin, S., Fan, L., Thiyagarajan, P. Application of SAXS and SANS in evaluation of porosity, pore size distribution and surface area of coal. *Int. J. Coal Geol.* **59**, 245 (2004).

9 Su, Z., Zhang, M., Lu, Z., Song, S., Zhao, Y., Hao, Y. Functionalization of cellulose fiber by in situ growth of zeolitic imidazolate framework-8 (ZIF-8) nanocrystals for preparing a cellulose-based air filter with gas adsorption ability. *Cellulose* **25**, 1997 (2018).

10 Lv, L., Han, X., Mu, M., Wu, X., Li, C. Templating metal-organic framework into fibrous nanohybrids for large-capacity and high-flux filtration interception. *J. Membr. Sci.* **622**, 119049 (2021).

11 Cheung, Y., Ma, K., Wasson, M., Wang, X., Idrees, K. B., Islamoglu, T., Mahle, J., Peterson, G. W., Xin, J. H., Farha, O. K. Environmentally benign biosynthesis of hierarchical mof/bacterial cellulose composite sponge for nerve agent protection. *Angew. Chem. Int. Ed.* **61**, e202202207 (2022).

12 Lýpez-Maya, E., Montoro, C., Rodrguez-Albelo, L. M., Cervantes, S. D. A., Lozano-Prez, A. A., Cens, J. L., Barea, E., Navarro, J. A. R. Textile/metal–organic-framework composites as self-detoxifying filters for chemical-warfare agents. *Angew.Chem. Int. Ed.* **54**, 6790 (2015).
